# Alteration of chromosome structure impacts gene expressions implicated in pancreatic ductal adenocarcinoma cells

**DOI:** 10.1186/s12864-024-10109-4

**Published:** 2024-02-23

**Authors:** Wenrui Han, Detong Shi, Qiu Yang, Xinxin Li, Jian Zhang, Cheng Peng, Fang Yan

**Affiliations:** 1 Yunnan Key Laboratory of Cell Metabolism and Diseases, Center for Life Sciences, School of Life Sciences, State Key Laboratory of Conservation and Utilization of Bio- resources in Yunnan, Yunnan University, 650500 Kunming, China; 2 Southeast United Graduate School, 650500 Kunming, China

**Keywords:** Non-genetic alterations, Chromosome accessibility, Three-dimensional genome structure, Pancreatic ductal adenocarcinoma, *LPAR1*

## Abstract

**Background:**

Pancreatic ductal adenocarcinoma (PDAC) is a lethal malignancy with a five-year survival rate of approximately 10%. Genetic mutations are pivotal drivers in PDAC pathogenesis, but recent investigations also revealed the involvement of non-genetic alterations in the disease development. In this study, we undertook a multi-omics approach, encompassing ATAC-seq, RNA-seq, ChIP-seq, and Hi-C methodologies, to dissect gene expression alterations arising from changes in chromosome accessibility and chromatin three-dimensional interactions in PDAC.

**Results:**

Our findings indicate that chromosomal structural alterations can lead to abnormal expressions on key genes during PDAC development. Notably, overexpression of oncogenes *FGFR2*, *FOXA2*, *CYP2R1*, and *CPOX* can be attributed to the augmentation of promoter accessibility, coupled with long-range interactions with distal elements. Additionally, our findings indicate that chromosomal structural alterations caused by genomic instability can lead to abnormal expressions in PDACs. As an example, by analyzing chromosomal changes, we identified a putative oncogenic gene, *LPAR1*, which shows upregulated expression in both PDAC cell lines and clinical samples. The overexpression is correlated with alterations in *LPAR1-*associated 3D genome structure and chromatin state. We further demonstrated that high LPAR1 activity is required for enhanced PDAC cell migration in vitro.

**Conclusions:**

Collectively, our findings reveal that the chromosomal conformational alterations, in addition to the well-known genetic mutations, are critical for PDAC tumorigenesis.

**Supplementary Information:**

The online version contains supplementary material available at 10.1186/s12864-024-10109-4.

## Background

Despite notable progress in therapeutic strategies, including immunotherapy, pancreatic cancer remains a formidable challenge within the field of oncology. Its five-year survival rate after diagnosis remains discouragingly low, approximately at 10% [1]. Delayed clinical presentation related to insidious symptoms, the lack of early detection strategies, the intricate biological attributes of pancreatic tumors, and the limited therapeutic options all contribute to the poor prognosis of this disease [2, 3].

Current investigations have emphasized that the genesis of pancreatic cancer predominantly arises from pancreatic intraepithelial neoplasia (PanIN), a precancerous lesion within the pancreas [4, 5]. In a complex interplay orchestrated by the accumulation of genetic mutations and exposure to a hostile microenvironment, PanIN lesions gradually advance into invasive malignancies [6–8]. Amidst the intricate tapestry of pancreatic cancer’s heterogeneity, a quartet of recurrent gene mutations has unfailingly assumed the spotlight, with *KRAS*, *TP53*, *SMAD4*, and *CDKN2A* standing as the four cardinal genes predominantly implicated [3, 6, 9].

In addition to gene mutations, chromosome structure plays a significant role in regulating gene expression [10–13]. Chromosome structure refers to the physical arrangement of chromosomes at various levels of organization, from the DNA double helix around histone proteins to higher-order structures. Recent studies have shown significant differences in higher-order structures between PDAC cell lines and their normal counterparts [14, 15]. For example, the tumor metastasis-associated gene *LIPC* is regulated by a specific enhancer-promoter loop in metastatic carcinoma cell lines [14]. Deletions of *SMAD4* and *CDKN2A* lead to changes in chromatin 3D structure, which affect the expression of oncogenes *MIR31HG*, *MYO5B*, among others [15]. Intriguingly, *KRAS* mutation alone is insufficient to drive tumor development, but rather changes in chromatin organization are required for tumorigenesis in a mouse model [16]. However, the relationship between chromosomal structural abnormalities and PDAC remains incomplete understanding.

In this study, we performed a comparative analysis of chromosome structure, including chromatin accessibility and chromatin 3D structures, in two types of PDAC cell lines (PANC-1 and BxPC-3) and an immortalized untransformed pancreatic epithelial cell line (H6C7, herein referred to as “normal cells” for clarity). We further explored the intricate interplay between the dysregulated expression of cancer-related genes and the landscape of chromosomal structural anomalies. Our findings suggest that chromosomal structural variations could affect the upregulation of oncogenic gene expression in pancreatic cancer cells. The methods utilized in this study have the potential to identify critical genes involved in malignancy progression.

## Methods

### Cell lines and cell culture

The human PDAC cell lines PANC-1 and BxPC-3 were sourced from the National Collection of Authenticated Cell Cultures (NCACC). Human pancreatic ductal epithelial cells H6C7 were procured from Hunan Fenghui Biotechnology. The cell lines were maintained in RPMI 1640 medium supplemented with 10% fetal bovine serum (FBS) and 1% penicillin and streptomycin (PS). Incubation of the cultures was conducted at 37℃ within a humidified atmosphere containing 5% CO2.

### ATAC-seq library preparation and data analysis

ATAC-seq libraries were prepared according to the established protocol [17] and then subjected to sequencing on the Illumina NovaSeq 6000 sequencer. The removal of adaptor-contaminated and low-quality reads was conducted using FastQC ver 0.11.9 and Trimmomatic ver 0.39 [18]. Subsequently, the resultant clean data sets underwent alignment to the human hg19 genome employing Bowtie2 ver 2.3.5.1 [19]. We employed the RPKM (Reads Per Kilobase per Million mapped reads) normalization method using deepTools ver 3.1.3 [20]. Identification of peaks corresponding to accessible chromatin regions was performed using MACS2 ver 2.1.1 [21] using the default parameters. Subsequently, we conducted differential analysis on peaks with the default parameters using DiffBind Package ver 3.4.11. Then we performed enrichment analysis on the classified peaks and annotated the peaks using ChIPseeker ver 1.30.3 [22].

### ChIP-seq library preparation and data analysis

ChIP assays were executed according to the established protocol [23], employing the following antibodies: CTCF (abcam, ab128873), RAD21 (abcam, ab992), and H3K4me3 (diagenode, C15410003). Then the ChIP-enriched DNA libraries were subjected to high-throughput sequencing using the Illumina NovaSeq 6000 sequencer. Stringent quality control was upheld, involving the elimination of adaptor-polluted and low-quality reads, facilitated by FastQC ver 0.11.9 and Trimmomatic ver 0.39 [18]. The clean data were then aligned to the human hg19 genome utilizing Bowtie2 ver 2.3.5.1 [19]. The identification of peaks was executed by MACS2 ver 2.1.1 [21] employing the default parameters.

### RNA-seq and data analysis

Total RNA extraction was carried out utilizing the TRIzol method. Then the RNA libraries were prepared following a standard protocol provided by Illumina. These libraries were subjected to high-throughput sequencing on the Illumina NovaSeq 6000 sequencer. After removal of adaptor-polluted and low-quality reads using FastQC ver 0.11.9 and Trimmomatic ver 0.39 [18], clean data were aligned to the human hg19 genome by Bowtie2 ver 2.3.5.1 [19]. For the assessment of gene expression levels, normalization was conducted using fragments per kilobase per million mapped fragments (FPKM) values,employing Stringtie ver 2.1.4 [24].. Differential expression analysis was then performed using DESeq2 ver 1.3.4.0 [25], enabling the identification of differentially expressed genes (DEGs). The criteria for differential genes were defined as a *p*-value < 0.05 and|log2FoldChange| > 0.5.

### Hi-C library preparation and data analysis

In situ Hi-C libraries were constructed in accordance with the established protocol [26] and then sequenced with Illumina NovaSeq 6000 sequencer. The acquired Hi-C data was subjected to alignment, correction, and processing using the software HiCHap ver 1.6 [27]. Principal component analysis (PCA) was performed on the correlation-based Hi-C heatmaps at a 500 kb resolution to identify A/B compartments. TAD calling was performed at the 40 kb Hi-C matrices. The boundary strength of TADs was displayed using deepTools ver 3.1.3. To elucidate intra-chromosomal interactions, we employed the NeoLoopFinder ver 0.2.3 [28] on the normalized contact matrices at the 40 kb resolution. Based on Hi-C data, genomic structure variations (SV) were identified using hic_breakfinder [29]. The copy number variations (CNV) were computed utilizing NeoLoopFinder ver 0.2.3.

### Quantitative reverse transcription PCR (RT-qPCR)

Total RNA was extraction from cell pellets using RNAiso Plus (TaKaRa) and subjected to DNase I treatment to eliminate any additional DNA. Then DNA-free RNA was transcribed into complementary (cDNA) utilizing the EasyScript® One-Step gDNA Removal and cDNA Synthesis SuperMix (TransGen Biotech) in accordance with the manufacture’s protocol. RT-qPCR was conducted using the SsoFast™ EvaGreen® Supermix (BIO-RAD) with primers listed in Table S2. The cycle threshold (Ct) value derived from the qPCR amplification was first normalized to the expression of the GAPDH and further transformed to relative expression in plots.

### Genomic amplification and sequencing

Total DNA was extraction from cell pellets using TIANamp Genomic DNA Kit (TIANGEN). Then the genomic fragments, including *PRDM15*, *ZBTB21*, and *MX1-ABCG1* were amplified using 2xTaq Master Mix (vazyme Bio-tech) with primers in table S2. PCR productions were purified and sequenced in both direction with BigDye Terminator Cycle Sequencing Kit on an ABI PRISM 3730 DNA analyser (Applied Biosystems). Sequences of each segment were proofread and assembled with DNASTAR 5.0 (DNASTAR Inc., Madison, WI, USA).

### Knockdown of *LPAR1* by siRNA

Transient transfections of siRNA were performed using GP-transfect-Mate (GenePharma, Suzhou, China) following the manufacturer’s instructions. The utilized siRNA sequence is provided in the supplemental information (Table S3). The efficiency of gene knockdown was assessed by measuring the RNA levels of *LPAR1* through RT-qPCR.

### Cell proliferation assay

Cells were seeded at a density of 5 × 10^3^ cells/well in 96-well plates. Then cells were treated with Ki16425 (Solarbio, IK0180) at varying concentrations, specifically 0µM, 1µM, 10 µM, and 20µM. In order to assess the cellular growth rate, a solution derived from the Cell Counting Kit-8 (CCK-8, GLPBIO) was added to each well at distinct times, namely 0, 10, 24 and 48 h. Following a 2-hour incubation period, the absorbance of the conditioned medium at 450 nm was measured. For cells subjected to *LPAR1* knockdown, the identical CCK-8 methodology was applied at 12, 16, 20, and 40 h post-seeding.

### Transwell migration assay

The transwell migration assay was conducted utilizing 8 μm PVDF inserts placed within a transwell chamber fitting into 24-well plates (Corning 3422). Each well was seeded with a population of 5 × 10^4^ cells in RPMI 1640 medium. The cells were permitted to migrate through the transwell inserts for a 16-hour period, into a medium supplemented with varying concentrations of Ki16425, specifically 0µM, 1µM, 10 µM, and 20µM. Transwell chambers were removed, followed by a thorough wash with PBS. The migrated cells were then fixed with 4% Paraformaldehyde (PFA) and subsequently stained with crystal violet. Cells that were not migrated through the insert were removed using a cotton swab. Migrated cells were visualized using a microscope. For cells subjected to *LPAR1* knockdown, the same methodology was applied without Ki16425.

### Scratch wound assay

Cells were seeded at a density of 1 × 10^6^ cells/well in 6-well plates. Scratch wounds were made with a 200ul pipette tip. Then each well was washed with PBS and cultured in RPMI 1640 with 2% FBS supplemented with varying concentrations of Ki16425, specifically 0µM, 1µM, 10 µM, and 20µM. Images were taken at time 0, 4, 6, 9, 12, 12, 24 and 30 h. For cells subjected to LPAR1 knockdown, the same methodology was applied without Ki16425.

## Results

### Chromatin accessibility alterations in PDAC cells

**Fig. 1:**
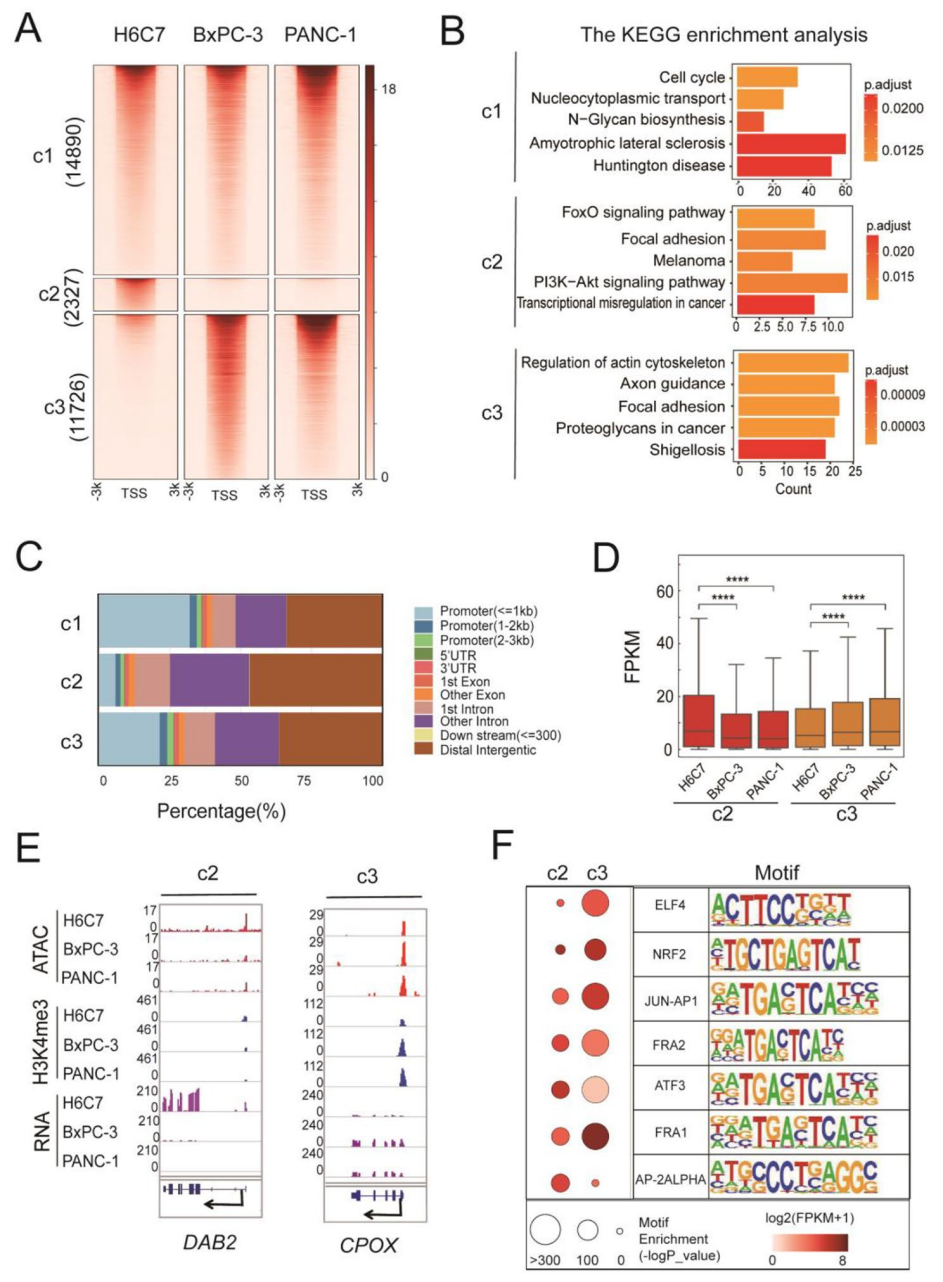
Changes in chromatin accessibility and its effect on gene expression in PDAC. **A** Heatmap of the ATAC-seq profiles. ATAC-seq peaks were classified into three clusters (C1, C2, C3) by K-means according to peak intensities in three cell lines. **B** Bar plot showing the enriched pathways of ATAC-seq peaks in each cluster, C1, C2, and C3. **C** Bar graphs showing the localizations of the ATAC-seq peaks in different clusters. **D** Expression of genes within three ATAC-seq peak clusters and H3K4me3 modification peak. **** means *p* value < 0.001. FPKM = fragments per kilobase million. **E** Examples for two ATAC-seq peak clusters in Fig. 1D. **F** Dot plots showing the enriched TFs motifs and the gene expressions (y-axis) in the different ATAC-seq peak clusters (x-axis). Circle size represents the motif enrichment, and the gradient red color indicates the relative gene expressions. FPKM = fragments per kilobase million

Dynamic chromatin opening allows gene-regulatory factors to interact with DNA elements to control gene expression in various cellular processes and cell fate decisions [30, 31]. To explore chromatin changes in PDAC cells, we compared chromatin accessibility between the normal and PDAC cells. We identified total 65,174 genomic regions with ATAC-seq peaks and classified into different clusters by peak intensities in three cell lines. Here, we focused on only three specific clusters. C1, peaks present in all three cell types, is mainly enriched in genes in cell cycle, and nucleocytoplasmic transport (Fig. 1A and B). C2, normal cell-specific peaks, is mainly enriched in the FoxO tumor suppressor and focal adhesion (Fig. 1A and B). C3, pancreatic cancer cell-specific peaks, is enriched in genes implicated in regulation of actin cytoskeleton, axon guidance, focal adhesion, and proteoglycans in cancer (Fig. 1A and B). Compared to C1, the accessibility regions of C2 and C3 are decreased in the proximal promoter area (Fig. 1C). Notably, C3 demonstrates an elevated activation ratio in proximity to the transcription start site compared to C2, signifying gene regulatory activation in PDAC cells.

Next, we analyzed the relationship between gene expression and chromatin state. As expected, genes close to ATAC-seq peaks associated with open chromatin state and H3K4me3 ChIP-seq peaks which mark active promoters were highly expressed (Fig. 1D). Furthermore, genes in C2 were highly expressed in the normal cells, but genes in C3 were highly expressed in the cancer cells (Fig. 1D and E).

We then applied motif enrichment analysis to cluster-specific accessible peaks to investigate the potential key transcription factors (TFs) in C2 and C3. We observed that large numbers of oncogenic TFs were enriched in C3, such as ATF3, ELF4, FRA1, FRA2, JUN-AP1, and NRF2 (Fig. 1F). While tumor suppressor TFs were enriched in C2, such as AP2ALPHA (Fig. 1F). Besides, these ATAC-seq signals showed consistency with RNA expression patterns, except for ATF3 (Fig. 1F).

Collectively, these findings demonstrate dynamic changes in accessible chromatin during PDAC tumorigenesis. Chromatin state and transcription factors play a crucial role in regulating changes in cancer-related transcriptional program.

### Global chromatin 3D structural changes in PDAC cells

**Fig. 2:**
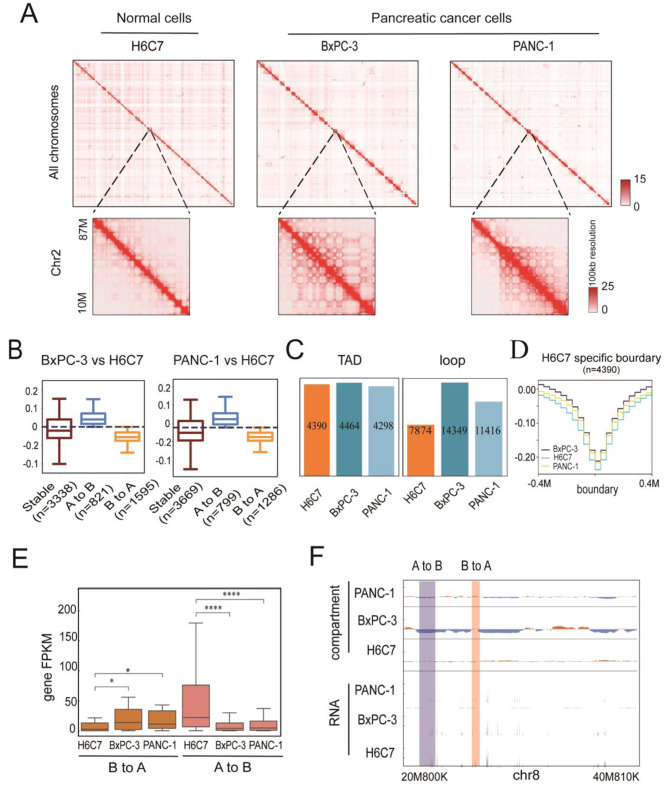
Changes in 3D genomic architecture and expression changes with compartment switching in PDAC. **A** Genome-wide Hi-C interaction maps of normal cells and pancreatic cancer cells. In the lower panels, enlargements of cis- and trans-interactions for Chr2 are shown. **B** The number of compartment switches of pancreatic cancer cells compared with normal cells. “A to B” means switched from compartment A to compartment B. “B to A” means the switched from compartment B to compartment A. **C** The number of TADs and loops in three cell lines. **D** Average insulation score shows no significant difference in three cell lines in H6C7 specific TAD boundaries. **E** Expression level of genes localized in different compartments. “B to A” means the compartment switched from B to A in BxPC-3 and PANC-1 compared with H6C7. “A to B” means compartment switched from A to B in BxPC-3 and PANC-1 compared with H6C7. **F** Visualization of one genomic fragment in Chr8 with compartment and RNA data

Chromatin 3D architecture, which refers to how chromatin is organized within the nuclei, can regulate gene induction and repression by controlling the physical access of transcriptional regulatory elements to DNA [11, 32]. Aberrant chromatin 3D structure can lead to developmental diseases and cancer [33, 34]. To investigate chromatin 3D structural changes in PDAC cells, we compared chromatin interaction maps of normal cells (H6C7) and PDAC cells (BxPC-3 and PANC-1). We found that both PDAC cell lines showed considerable chromatin 3D structural alterations from the normal cell line (Fig. 2A). For example, significant rearrangements were observed at the 10–87 Mb region of chromosome 2 (Fig. 2A). Compared to the normal cells, PDAC cells showed increased chromosomal interactions in the region.

We then investigated chromatin 3D organization changes at compartment, topologically associating domain (TAD), and chromatin loop levels. At the compartment level, compared to normal H6C7, BxPC-3 and PANC-1 genomes showed 41.99% (A compartment to B compartment switch, 14.27%, B-to-A, 27.72%) and 36.21% (A-to-B, 13.86%, B-to-A, 22.35%) compartment switching, respectively (Fig. 2B). Moreover, clear loop rearrangement events occur in the PDAC cells. The number of loops increased dramatically in the PDAC cells (Fig. 2C), corresponding to the increased chromosomal interactions observed in genome-wide Hi-C interaction maps. However, no obvious difference in TAD numbers and boundary strength were found between normal cells and the PDAC cells (Fig. 2C and D).

We further analyzed RNA-seq data to investigate gene expression pattern alterations associated with high-order genomic changes in the PDAC cells. As predicted, genes in the A compartments tend to express at higher levels than those in the B compartments (Fig. 2E). In most cases, gene expressions were upregulated when a compartment switches from B to A, while gene expressions were downregulated when a compartment switches from A to B (Fig. 2E and F).

These findings suggest that high-order chromatin structures undergo marked changes during pancreatic carcinogenesis. The observed compartment switches in the PDAC cells likely played an important role in modulating gene expression changes.

### Upregulation of oncogenes correlated with loops/TADs reprograming and promoter opening in PDAC cells

As previously detected, there exists a compelling association between alterations in chromosome structure and abnormal gene expressions. Indeed, we found that changes in 3D chromatin structures and chromatin state in the PDAC cells are linked to the upregulation of oncogene expressions.

**Fig. 3:**
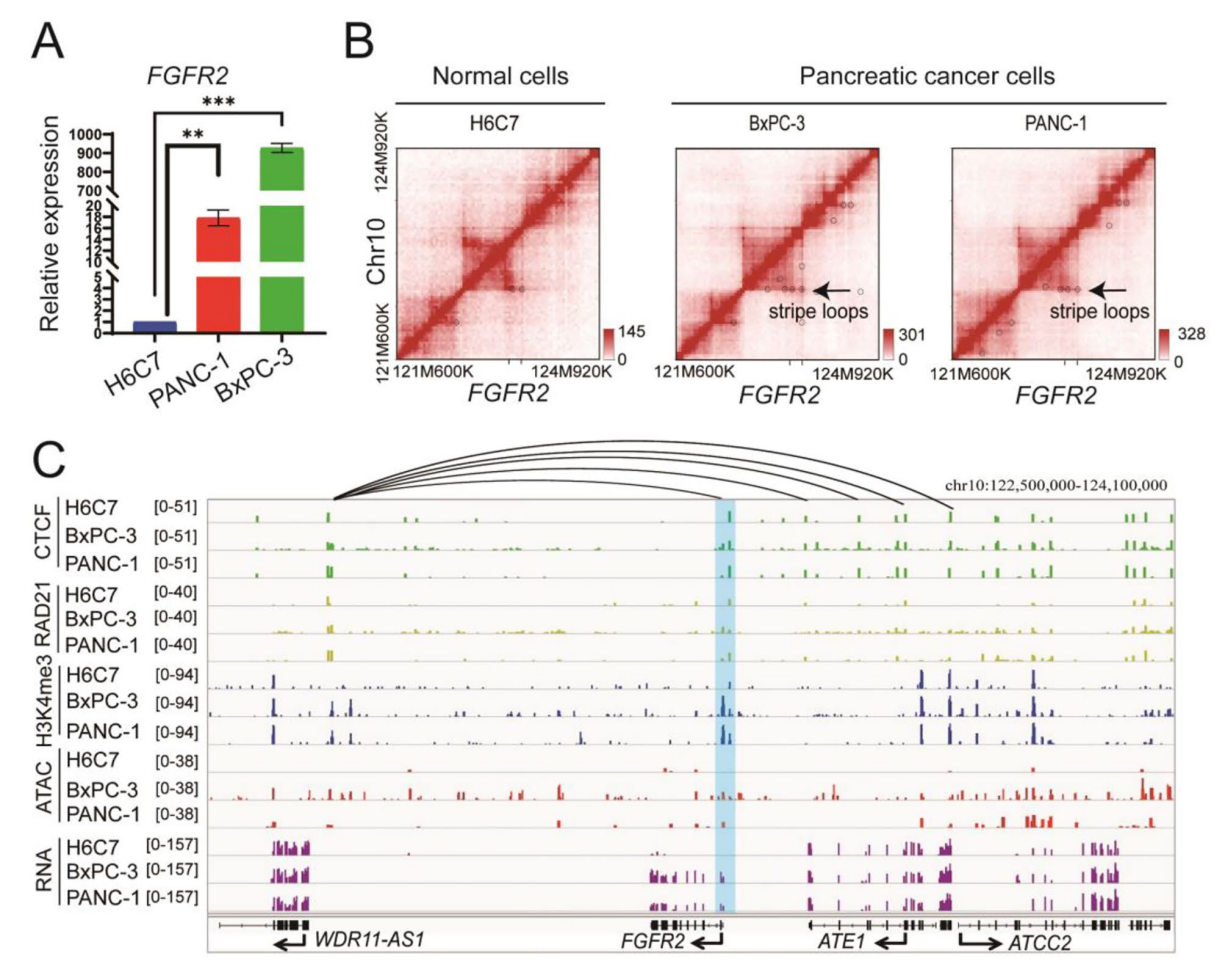
Cancer-specific chromatin structure upregulated *FGFR2* via reshaping loops and opening chromatin in PDAC. **A** Expression of *FGFR2* in three cell lines. ** means *p* value < 0.001. *** means *p* value < 0.0005. **B** Hi-C interaction maps showed stripe loops formation involving *FGFR2* in PANC-1 and BxPC-3. **C** ChIP-seq (CTCF, Rad21, H3K4me3), ATAC-seq, and RNA-seq tracks surrounding the *FGFR2* in three cell lines. The box with blue color showed the location of promoter of *FGFR2*

For instance, the oncogene *FGFR2* displays significant upregulation within cancer cells (Fig. 3A). Intriguingly, it is located in a genomic region with the newly formed stripe loops (Fig. 3B), a loop anchor interacts with entire domains at high frequency [35]. These stripe loops enhance the direct interaction among several regions in 122.70 Mb-123.73 Mb of chromosome 10 (Fig. 3C), allowing for physical connections between the promoter region of *FGFR2* and distant elements. This intricate network of physical interactions facilitates the coordination required for precise gene regulation. Intriguingly, this phenomenon coincides with a transition of the chromosome from a closed conformation to an open state, enabling transcription factors to bind easily to the promoter region of *FGFR2* and thereby facilitating gene expression. The presence of H3K4me3 modification further underscores the activity of the promoter region, solidifying the notion of active gene transcription.

Similar patterns of transitive loops or loop stripes were observed for additional oncogenes, such as *FOXA2*, *CYP2R1*, and *CPOX*, within their respective genomic domains (Fig. S1-S3). This synchronous alteration signifies a broader regulatory network that orchestrates the activation of these oncogenes through a shared mechanism involving 3D chromatin remodeling and chromatin state transitions.

### Reprogramed chromatin structures induced by genomic deletions associated gene expression changes in PDAC cells

Structural variations (SVs) can influence the 3D genome structure by disrupting the higher-order structure of chromosomes [36]. Based on our Hi-C data, we identified chromosome structural alterations caused by structural variations (Table S4), including two well-known genes crucial in the occurrence of pancreatic cancer: *SMAD4* and *CDKN2A.* Correspondingly, SVs have altered the spatial relationships among chromosomes, accompanied by the emergence the neo-loops (Table S5). Integrating RNA-seq, ATAC-seq, and ChIP-seq results, we have uncovered a novel deletion that merits our attention.

**Fig. 4:**
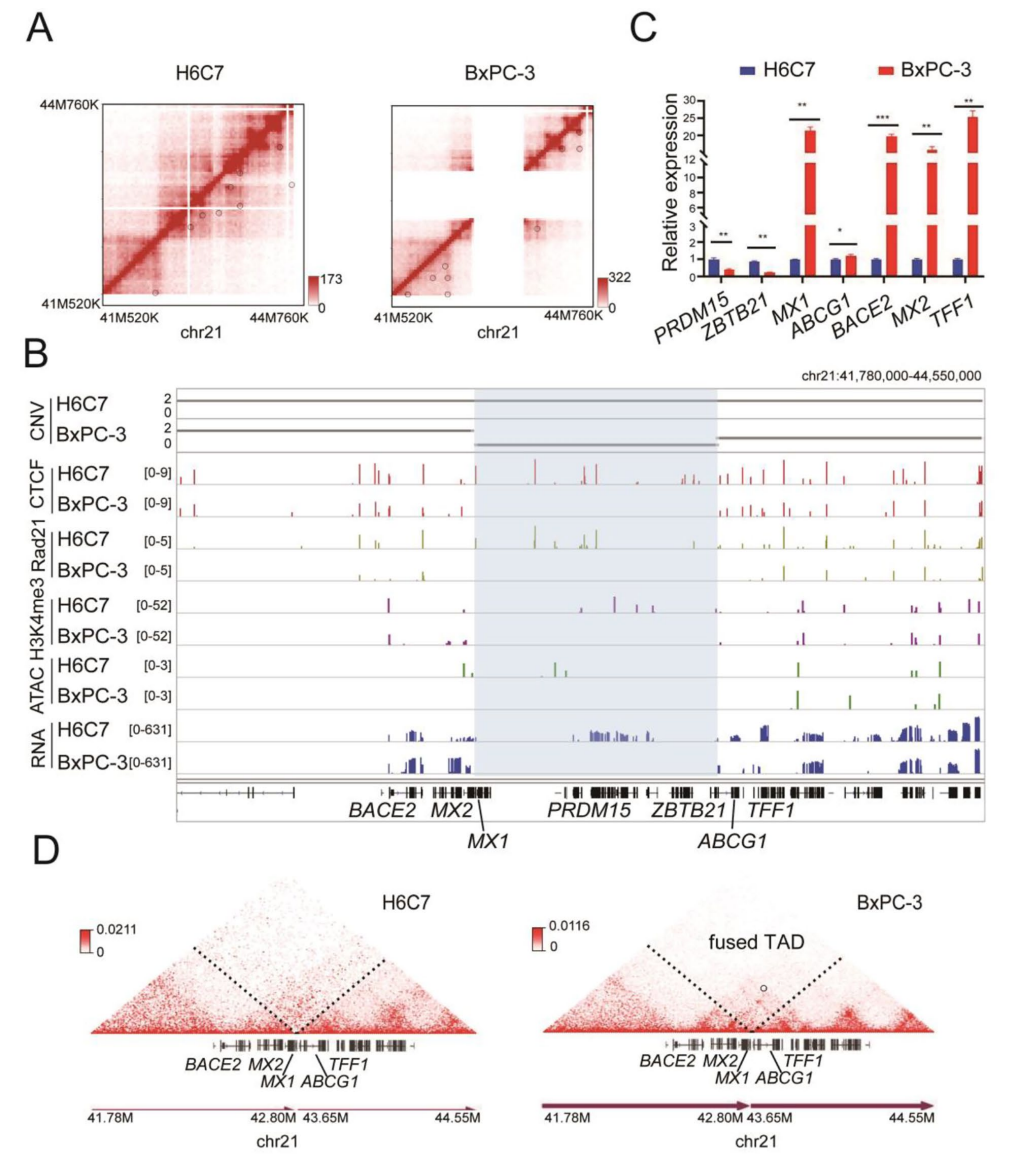
Dysregulation of cancer-related genes through TAD fusion associated with deletion. **A**: Hi-C interaction maps showed deletion on Chr21 in BxPC-3. **B**: Copy number (CN), ChIP-seq (CTCF, Rad21, H3K4me3), ATAC-seq, and RNA-seq tracks surrounding the translocation locus in H6C7 and BxPC-3. The box with blue color showed the location of deletion. **C**: Expression of *PRDM15*, *ZBTB21, MX1, ABCG1, BACE2, MX2, and TFF1* in H6C7 and BxPC-3. * means *p* value < 0.05. **D**: Triangle heatmap showing the new interaction on both sides of the deletion in BxPC-3

The deletion located between 42.80 Mb-43.65 Mb in chromosome 21 resulted in absence of genes in the deleted region, including *TMPRSS2, LINC00111, LINC00479, RIPK4, PRDM15, C2CD2, SNORA91, ZBTB21, ZNF295-AS1*, and *UMODL1* in BxPC-3 cells (Fig. 4A). Among these genes, *PRDM15* and *ZBTB21* were highly expressed in the normal cells (Fig. 4B and C). In addition, the deletion generated a *MX1-ABCG1* gene fusion, which was upregulated in the PDAC cells (Fig. 4B and C). Genomic amplification further validates the deletion and fusion (Fig. S4). We further observed that some adjacent genes, such as *BACE2, MX2, and TFF1* were also upregulated in the PDAC cells (Fig. 4B and C). Moreover, interactions between chromatin segments on both sides of the deletion was enhanced, forming a fused new TAD and neo-loops (Fig. 4D). The TAD includes *BACE2, MX2, MX1-ABCG1*, *and TFF1*, which showed upregulated expression as expected. Enhanced chromosomal accessibility and higher level of H3K4me3 modifications at their respective promoter regions were also detected (Fig. 4B). These results suggest that the reprogrammed chromatin structures likely contribute to the altered gene upregulation.

### Chromosome structure changes in PDAC caus *e LPAR1* upregulation

Our investigation into abnormal RNA expressions and associated chromosome structure changes has led us to identify potential genes involved in tumorigenesis. By integrating data from Hi-C, RNA-seq and ATAC-seq, we have identified 72 genes that display significant upregulation in two PDACs, accompanied by open chromatin structures and formation of novel loops (Table S6). The four oncogenes previously described, *FGFR2, FOXA2, CYP2R1, and CPOX*, are also included in this list. The top five genes, arranged in descending order based on expression differences, are *FABP5, GLUL, FLI1, ANO1*, and *LPAR1*. Through a comprehensive analysis of gene expression and epigenetic signals within these genes and their neighboring counterparts in genome, we ultimately selected *LPAR1* as a candidate oncogene for further experimental investigation.

**Fig. 5:**
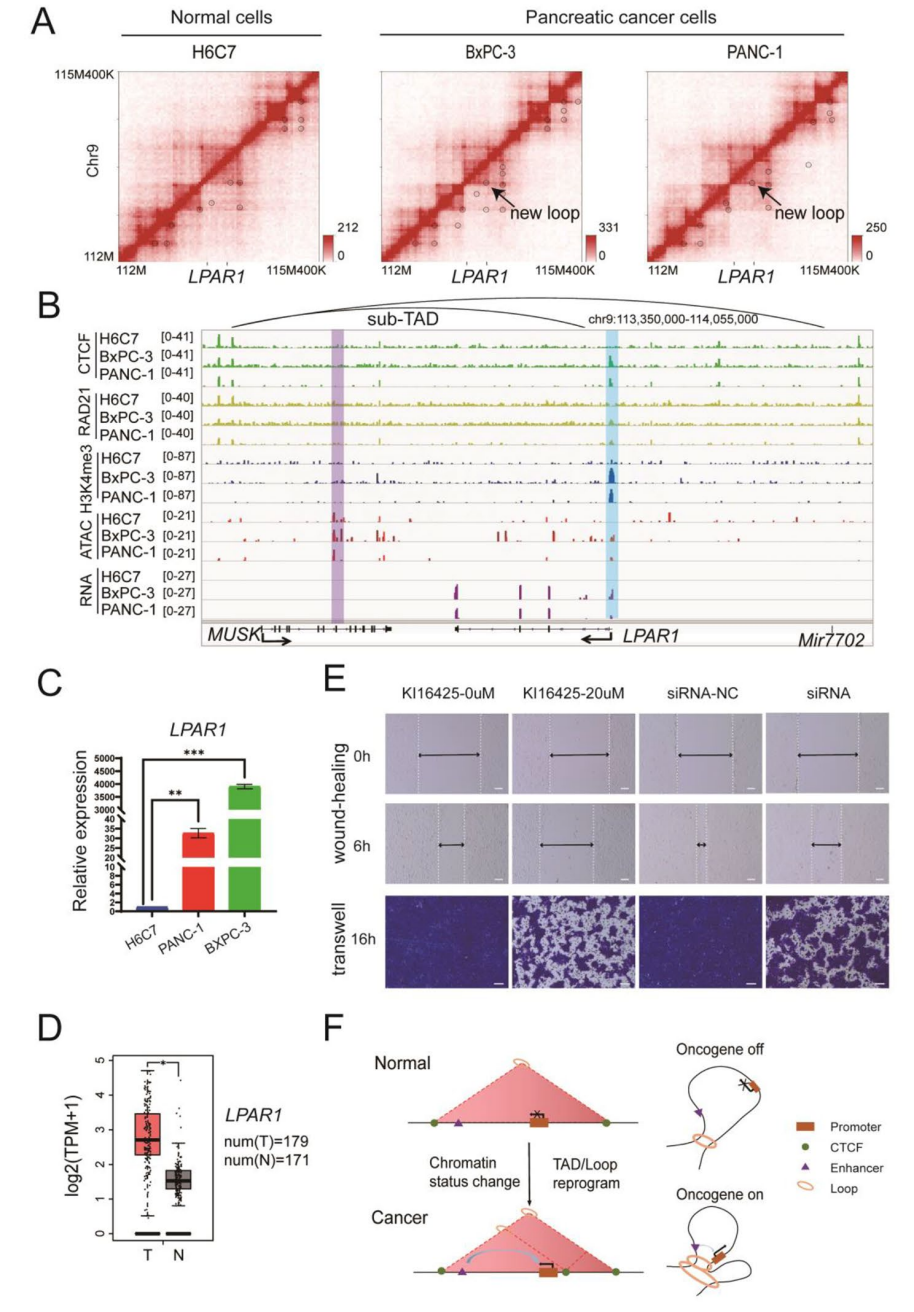
*LPAR1*, upregulated by chromatin structure changes in pancreatic cancer cells, promotes cell migration. **A** Hi-C interaction maps surrounding the *LPAR1* locus in three cell lines. **B** ChIP-seq (CTCF, Rad21, H3K4me3), ATAC-seq, and RNA-seq tracks surrounding the *LPAR1* locus in three cell lines. The box with blue color showed the location of promoter of *LPAR1*. The box with purple color showed the putative cis-regulatory element (possible enhancer). **C** RT-qPCR performed on *LPAR1* in three cell lines. ** means *p* value < 0.001. *** means *p* value < 0.0005. **D** Expression of *LPAR1* in pancreatic cancer and normal tissues from TCGA. T = tumor tissue. N = normal tissue. TPM = transcript per million. num means number of samples. * means *p* value < 0.01. **E** The scratch wound assay (upper panel) and the transwell test (lower panel) showed that Ki16425 (the antagonist for LPAR1) and knockdown of *LPAR1* markedly inhibited the migration of pancreatic cancer cells BxPC-3. Scale bar = 100 μm. **F** Schematic diagram showing that chromatin structure changes could switch oncogene’s expression during the progression of PDAC

*LPAR1* is located in chr9q31.3 with clear changes in 3D genome structure and chromatin state (Fig. 5A and B). A new loop was formed in this region, divided the original TAD in normal cells into two sub-TADs in cancer cells. ChIP-seq data indicated CTCF enrichment in the new loop anchor in PDAC cells and a putative cis-regulatory element(possible enhancer) was detected in the newly formed sub-TAD. Furthermore, H3K4me3 modification was strongly upregulated at the promoter region of *LPAR*1 in the PDAC cells compared to the normal cells. ATAC-seq data indicated the higher chromatin accessibility in the *LPAR1* locus in the PDAC cells than that in the normal cells.

We then investigated effects of these changes on gene expression in the PDAC cells. RNA-seq data showed that *LPAR1* was highly expressed in PDAC cells but not expressed in normal cells (Fig. 5B). The *LPAR1* transcription upregulation was confirmed using RT-qPCR (Fig. 5C). Overexpression of *LPAR1* in clinical PDAC cells was validated from published data in TCGA database (Fig. 5D). These findings suggest that the newly formed loop and opened chromatin might be associated with the activation of *LPAR*1 expression in PDAC cells.

Furthermore, we examined whether LPAR1 upregulation was a causal factor for PDAC cell proliferation or migration. Firstly, we used a known *LPAR1* inhibitor, Ki16425 to inhibit *LRAR1*/*LPA*-induced activation of p42/p44 MAPK [37, 38]. Blocking LPAR1 activity did not affect PDAC proliferation (Fig. S5A). Nevertheless, inhibiting LPAR1 activity significantly decreased PDAC cell migration, as evidenced by transwell assays and scratch wound assays (Fig. 5E, Fig. S6). Subsequently, we performed LPAR1 knockdown experiments utilizing small interfering RNA (siRNA). Proliferation and migration assays were executed, yielding consistent outcomes with the inhibition of LPAR1 activity using Ki16425 (Fig. S5B, Fig. 5E, Fig. S6). These findings collectively suggest the potential involvement of LPAR1 in PDAC metastasis.

Our investigation revealed a critical mechanism responsible for the upregulation of oncogenes in PDAC cells (Fig. 5F). In normal cells, the expression of oncogenes is restrained, partially attributed to the chromatin’s compacted state. In contrast, PDAC cells exhibit a distinct remodeling in chromatin structure, facilitating the activation of oncogene expressions.

## Discussion

Genetic mutations have traditionally been regarded as primary catalysts driving the onset and progression of cancer. Nevertheless, emerging evidence underscores the significance of non-genetic sequence alterations on malignancy progression [16, 39]. Chromatin structure, comprising chromatin accessibility and chromatin 3D structure, exerts a pivotal influence on transcriptional processes by intricately modulating the physical accessibility of transcriptional regulatory factors to DNA. Hence, the investigation of chromatin structure modifications within cancer cells not only enhances our comprehension of tumorigenesis but also holds the potential to facilitate the development of innovative therapeutic approaches.

In this study, our investigation yielded significant insights into the non-genetic characteristics of PDAC cells. The notable alterations observed encompassed both the chromatin state and 3D architecture within PDAC cells. Interestingly, the signaling pathway “Proteoglycans in Cancer,” recognized for its involvement in malignancy-associated processes [40], emerged as notably enriched within PDAC cell-specific accessible regions. This enrichment suggests a potential role of this pathway in driving the distinct characteristics of PDAC cells. Furthermore, the analysis of PDAC cell-specific chromatin accessible types revealed a significant enrichment of binding motifs for transcription factors associated with PDAC progression, such as NRF2 [41], JUN-AP1 [42], and ATF3 [43]. At the higher-order structure, PDAC cells demonstrated heightened chromatin interactions, signifying anomalous co-expression correlations among interacting gene pairs. Approximately one-third of gene loci in PDAC cells exhibited transitions in chromatin compartments. Moreover, these transitions correlated with changes in the expression levels of linked genes. This intriguing connection suggests a dynamic interplay between chromatin organization and gene expression in the context of PDAC, in accordance with previous findings [15]. These findings collectively signify that the remodeled chromatin landscape in PDAC may profoundly shape the expression profile underlying carcinogenesis.

Specifically, we made observations on the regulatory mechanisms controlling the expression of particular oncogenes within PDAC cells. We detected the existence of transitive loops or loop stripes in genomic regions linked to specific oncogenes within PDAC cells. Additionally, these oncogenes were observed to exhibit an activated chromatin state. One of the notable cases we explored was FGFR2, a tyrosine kinase receptor that plays a pivotal role in propagating downstream signaling pathways upon its interaction with Fibroblast Growth Factor (FGF). This receptor has garnered considerable attention due to its involvement in diverse biological processes, ranging from normal organ development to tumorigenesis [44]. In the broader landscape of cancer research, FGFR2 has been implicated in various tumors, with well-documented genetic aberrations such as gene amplifications, mutations, and structural variants [45]. These genetic perturbations often result in altered protein expression levels or variations in protein structure, contributing to the aberrant cellular behavior associated with tumorigenesis. Here, our study introduces a fascinating departure from the established genetic explanations for FGFR2 overexpression. Specifically, we have identified and reported a non-genetic regulatory mechanism that elucidates the phenomenon of FGFR2 overexpression in PDAC cells. Our findings unveil a novel dimension of gene regulation, wherein newly formed stripe loops play a significant role in enhancing physical interactions between the opened promoter of *FGFR2* and distant elements. These interactions create a microenvironment that facilitates the initiation of gene expression processes. Importantly, our findings emphasize the interplay of multiple factors in driving gene expression. It is noteworthy that both the formation of physical contacts and the establishment of an open chromatin state are integral contributors to the expression of target genes. This highlights the complexity of regulatory landscape and necessity to consider a holistic perspective when dissecting mechanisms governing gene expression in PDAC cells.

Chromatin structure changes were found to be coupled to genomic instability in cancer cells [46]. We detected a deletion region on chromosome 21 in BxPC-3 cells, resulting in inactivation of genes such as *PRDM15*, *C2CD2*, and *ZBTB21*, and upregulation of *MX1-ABCG1* (a newly fused gene), *BACE2*, *MX2*, and *TFF1.* The gene expression changes were likely resulted from a new fusion TAD and multiple new loops in the PDAC cells. The *PRDM* family members play role in cancer suppression, while *ABCG1*, *BACE2*, and *TFF1* are implicated in tumor formation in various studies [47–50]. Thus, in addition to direct gene deletion, fission and fusion, emergence of new chromosomal structures also alters expression of the structures associated genes.

Our exploration into the interplay between chromatin structure changes and gene expression abnormalities has led us to identify a potential candidate oncogene, *LPAR1*, in PDAC. Initially, we identified a striking contrast in the chromatin state of the *LPAR1* region between normal and PDAC cells. In normal cells, the chromatin surrounding the *LPAR1* gene appeared to be tightly packed, signifying a closed state. In contrast, our observations within PDAC cells unveiled a distinctively different scenario. The promoter region of the *LPAR1* gene exhibited an active chromatin state, facilitated by the formation of loops connecting it to distal cis-regulatory emelemt. This structural alteration provided a conducive environment for aberrant high expression of *LPAR1*, underscoring the pivotal role of chromatin structure in gene regulation. While our current study cannot conclusively prove that genomic structure changes directly cause the abnormal gene expression. The direct causal relationship necessitates a more in-depth investigation. But importantly, upregulation of *LPAR1* transcription was also observed in 179 pancreatic cancer patient samples, suggesting it is a common alteration in PDAC. LPAR1, a member of the lysophosphatidic acid receptor family, has been reported in previous studies due to its role in tumor cell proliferation, survival, invasion, and metastasis [51, 52]. Particularly noteworthy are its roles in central nervous system disorders and diseases, further emphasizing its multifaceted significance in cellular processes [53]. Our in vitro experimentation demonstrated that LPAR1 indeed plays a role in the migration of PDAC cells. This insight suggests a potential involvement of LPAR1 in the metastatic behavior of PDAC cells, which aligns with the previous understanding of LPAR1’s influence on pancreatic tumor progression [54, 55] and highlights its potential as a clinical and biological marker of PDAC.

## Conclusion

In this study, our findings underscore the crucial role of chromatin structure in influencing gene expression driving PDAC. Given the widespread use of BxPC-3 and PANC-1 in various studies, our research has the potential to offer valuable insights to the broader scientific community. Nevertheless, it is imperative to validate these findings in alternative cell lines or clinical samples to enhance the comprehensiveness of our understanding of the role of epigenetics in PDAC.

### Electronic supplementary material

Below is the link to the electronic supplementary material.

Supplementary Material 1

Supplementary Material 2

## Data Availability

All raw and processed sequencing data generated in this study have been submitted to the National Genomics Data Center (https://ngdc.cncb.ac.cn/?lang=zh) under accession number HRA005763. The data analysis script is available at https://github.com/shidetong/Pancreatic-cancer-scripts.
