# Supplementary material for: Alteration of chromosome structure impacts gene expressions implicated in pancreatic ductal adenocarcinoma cells
Source: BMC Genomics. 2024 Feb 23;25:206. doi: 10.1186/s12864-024-10109-4 (PMC10885383; doi:10.1186/s12864-024-10109-4)
Supplement: Supplementary file 2 — Supplementary Material 2 [file 12864_2024_10109_MOESM2_ESM.docx]

**Supplementary figures**

**Fig. S1** **Cancer-specific chromatin structure upregulated *FOXA2* via reshaping loops in PDAC**. **A** Hi-C interaction maps showed loops formation involving *FOXA2* in PANC-1 and BxPC-3. **B** RNA-seq, ATAC-seq and ChIP-seq (H3K4me3, Rad21, CTCF) tracks surrounding the *FOXA2* in three cell lines.

**Fig. S2 Cancer-specific chromatin structure upregulated *CYP2R1* via reshaping loops in PDAC**. **A** Hi-C interaction maps showed loops formation involving *CYP2R1* in PANC-1 and BxPC-3. **B** RNA-seq, ATAC-seq and ChIP-seq (H3K4me3, Rad21, CTCF) tracks surrounding the *CYP2R1* in three cell lines.

**Fig. S3** **Cancer-specific chromatin structure upregulated *CPOX* via reshaping loops in PDAC**. **A** Hi-C interaction maps showed stripe loops formation involving *CPOX* in PANC-1 and BxPC-3. **B** RNA-seq, ATAC-seq and ChIP-seq (H3K4me3, Rad21, CTCF) tracks surrounding the *CPOX* in three cell lines.

**Fig. S4. Genomic amplification of fused gene and genes in deletion region showed in figure 3**. **A**: Gel electrophoresis of PCR amplification products. M = DNA marker. Lines 1-3 are PCR productions of fused genes *MX1-ABCG1* with three primers in Table S2 for cell lines BxPC-3. Lines 4 and 6 are PCR productions of *ZBTB21* in H6C7 and BxPC-3, separately. Lines 5 and 7 are PCR productions of *PRDM15* in H6C7 and BxPC-3, separately. **B**: The schematic diagram of fused gene *MX1-ABCG1*. The fused sequence was detected by Sanger sequencing.

**Fig. S5**. **Antagonization of LPAR1 activity and knockdown of *LPAR1* didnot impede PDAC proliferation.** **A**: Cell inhibition rate was calculated in cell lines BxPC-3 and PANC-1 by antagonization of LPAR1 activity. * means p value < 0.05. ** means p value < 0.001. ns = not significant. **B**: Cell inhibition rate was calculated in cell lines BxPC-3 and PANC-1 by knockdown of *LPAR1*. * means p value < 0.05. ns = not significant.

**Fig. S6**. **LPAR1 promotes cell migration of PANC-1.** **A** The scratch wound assay (upper panel) and the transwell test (lower panel) showed that Ki16425 (the antagonist for LPAR1) and knockdown of *LPAR1* markedly inhibited the migration of pancreatic cancer cells PANC-1. Scale bar = 100μm.
